# A novel multi-functionalized multicellular nanodelivery system for non-small cell lung cancer photochemotherapy

**DOI:** 10.1186/s12951-021-00977-3

**Published:** 2021-08-14

**Authors:** Yongtai Zhang, Qing Xia, Tong Wu, Zehui He, Yanyan Li, Zhe Li, Xuefeng Hou, Yuanzhi He, Shuyao Ruan, Zhi Wang, Jia Sun, Nianping Feng

**Affiliations:** 1 Department of Pharmaceutical Sciences, Shanghai University of Traditional Chinese Medicine, Shanghai, 201203 China; 2 Teaching Experiment Center, Shanghai University of Traditional Chinese Medicine, Shanghai, 201203 China

**Keywords:** Photodynamic therapy, Red blood cell, Biomimetic, Nanoparticles, Liposomes, Tumor

## Abstract

**Background:**

A red blood cell membrane (RBCm)-derived drug delivery system allows prolonged circulation of an antitumor treatment and overcomes the issue of accelerated blood clearance induced by PEGylation. However, RBCm-derived drug delivery systems are limited by low drug-loading capacities and the lack of tumor-targeting ability. Thus, new designs of RBCm-based delivery systems are needed.

**Results:**

Herein, we designed hyaluronic acid (HA)–hybridized RBCm (HA&RBCm)-coated lipid multichambered nanoparticles (HA&RBCm-LCNPs) to remedy the limitations of traditional RBCm drug delivery systems. The inner core co-assembled with phospholipid-regulated glycerol dioleate/water system in HA&RBCm-LCNPs met the required level of blood compatibility for intravenous administration. These newly designed nanocarriers had a honeycomb structure with abundant spaces that efficiently encapsulated paclitaxel and IR780 for photochemotherapy. The HA&RBCm coating allowed the nanocarriers to overcome the reticuloendothelial system barrier and enhanced the nanocarriers specificity to A549 cells with high levels of CD44. These properties enhanced the combinatorial antitumor effects of paclitaxel and IR780 associated with microtubule destruction and the mitochondrial apoptotic pathway.

**Conclusions:**

The multifunctional HA&RBCm-LCNPs we designed expanded the functionality of RBCm and resulted in a vehicle for safe and efficient antitumor treatment.

**Graphical abstract:**

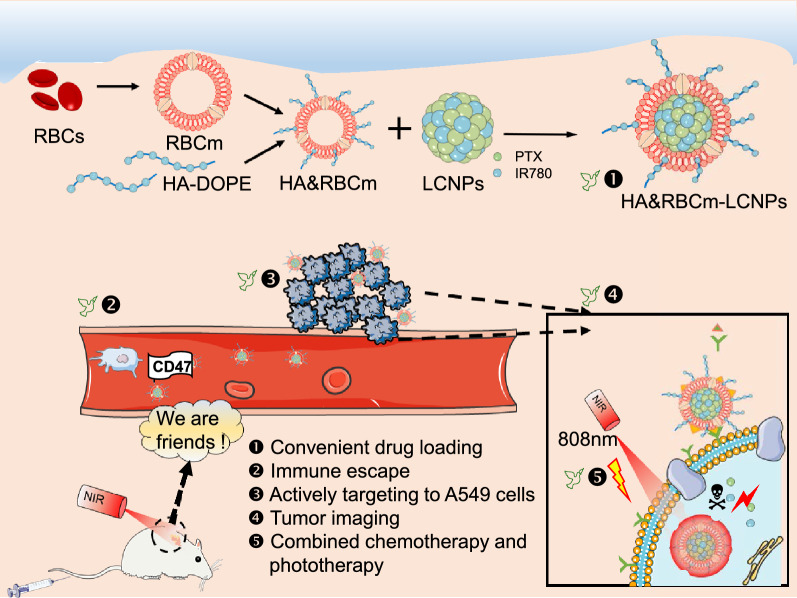

**Supplementary Information:**

The online version contains supplementary material available at 10.1186/s12951-021-00977-3.

## Background

Lung cancer has been associated with the highest mortality rate among all cancers in 2020 worldwide [1]. Currently, pharmacotherapy remains the primary treatment strategy against lung cancer. However, owing to difficulties in the delivery of traditional drug preparations to the tumor tissue, medications are frequently administered, thereby increasing the incidence of toxic side effects.

Nanocarriers are widely employed in tumor therapy research for their ability to accumulate in tumors and their controlled drug-release properties [2]. Among various nanocarriers, biomimetic nanoparticles have received great attention [3–5]. For successful drug delivery to tumor tissues, a drug construct must not be eliminated by the reticuloendothelial system (RES) and should achieve prolonged circulation in the blood [6, 7]. A common method for extending the retention time of therapeutics in the blood is through the use of stealth nanoparticles camouflaged with polyethylene glycol (PEG), which increases the hydrophilicity of the constructs and allows escape from RES phagocytosis [8]. However, although PEGylation prolongs the circulation of nanoparticles, PEG overexposure leads to anti-PEG IgG production, which induces humoral immune response and RES phagocytosis [9, 10]. As this is typically followed by an accelerated blood clearance, the clinical application of PEGylated nanocarriers remains a challenge [11].

In recent years, cell membrane-derived biomimetic nanocarriers have been used to extend the circulation and to facilitate tumor accumulation of drugs [12]. These nanocarriers are derived from bacteria, cancer cells, stem cells, lymphocytes, platelets, white blood cells, and red blood cells (RBCs). RBC membranes (RBCm) have been used as nanocarriers since the mid-1990 s [13]. Compared to PEGylated nanoparticles, RBCm have various advantages, including ease of availability, an endogenous nature, and low immunogenicity [14–17]. However, RBCm lack tumor-specific adhesion molecules and have low drug-loading capacity. The reliability of passive tumor-targeted drug delivery using nanoparticles with enhanced permeability and retention (EPR) remains controversial [18]. Therefore, RBCm nanocarriers require structural modifications to increase their ability to deliver drugs into tumor tissues [19].

**Fig. 1:**
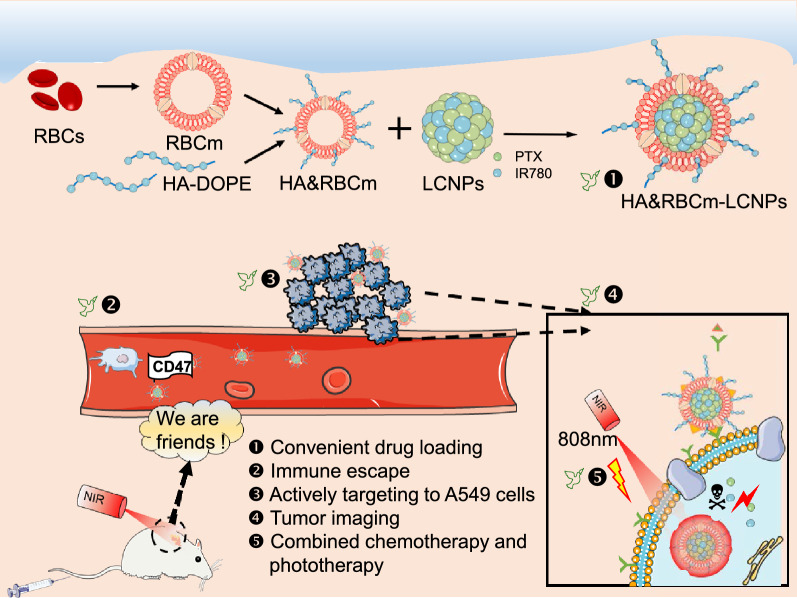
Schematic illustration of the preparation of HA&RBCm-LCNPs, its prolonged circulation in the blood, and its ability to actively target A549 cells for photochemotherapy of tumors

Lipid multi-chamber nanoparticles (LCNPs), such as cubosomes, multilayered liposomes, and multichamber liposomes have been widely used as delivery vehicles for various drugs due to their excellent drug-loading properties [20–22]. In this study, RBCm-coated phospholipid-mediated LCNPs (RBCm-LCNPs) possessing honeycomb or spongy structures biocompatible with intravenous administration, were used as carriers for co-loading paclitaxel (PTX) and the near-infrared dye, IR780, to achieve extended circulation and combined chemotherapy and phototherapy. To enhance the tumor accumulation of the vector based on the high expression of the CD44 protein in human non-small cell lung cancer cells (A549), hyaluronic acid (HA), a natural receptor for CD44 that exerts good biocompatibility, biodegradability, and non-immunogenicity properties, was selected to hybridize RBCm (HA&RBCm) [23–25]. In the present study, we investigated the circulation, the mechanism, and the potential value of this new drug delivery system in combinatorial tumor photochemotherapy (Fig. 1).

## Results and discussion

### Characterization of the preparations

**Fig. 2:**
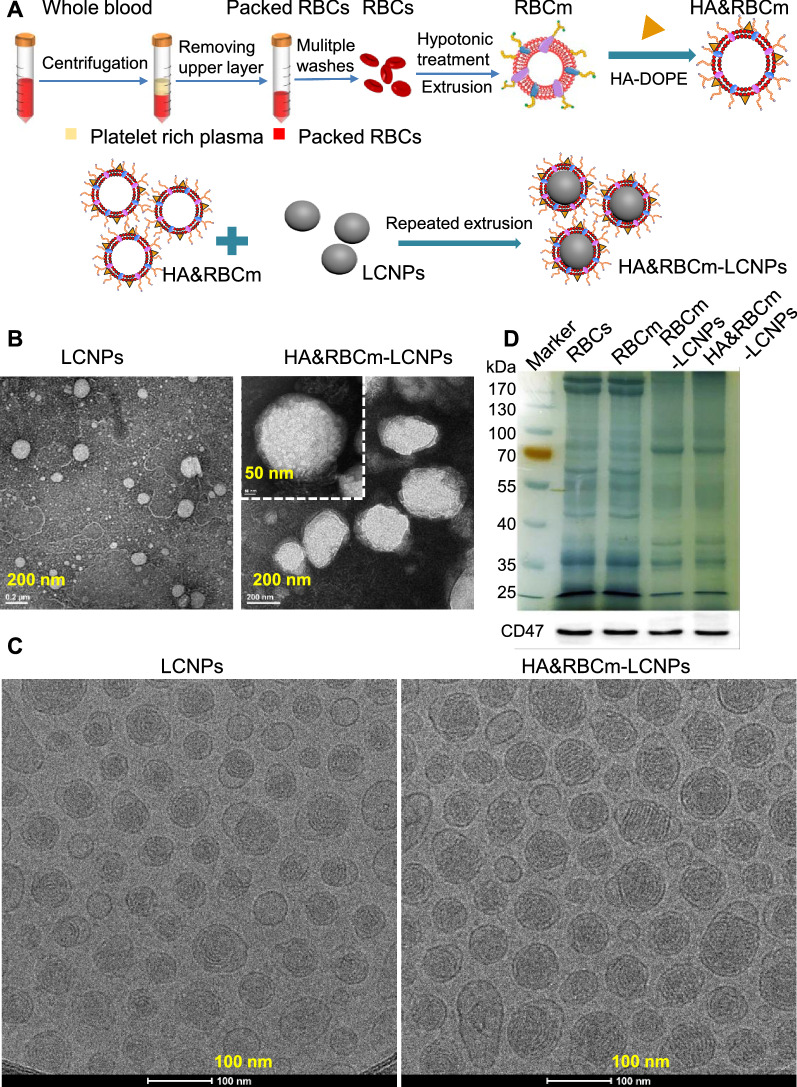
Characteristics of PTX/IR780-loaded nanocarriers. **A** Schematic diagram of the HA&RBCm-LCNP production process. **B** Transmission electron micrographs. **C** Cryo-transmission electron micrographs. **D** Sodium dodecyl sulfate polyacrylamide gel electrophoresis (SDS-PAGE) and western blotting analysis

In recent years, the most studied system for LCNPs formulation has been the glycerol monooleate/water system, which is valued for its remarkable stability, simple and green manufacturing process, and low material cost. However, unsaturated monoglyceride causes hemolysis in animals; thus, this system is not suitable for intravenous administration [26, 27]. Therefore, in this study, we prepared LCNPs using a biocompatible, phospholipid-regulated glycerol dioleate/water system suitable for injection (Fig. 2).

As the normal thickness of RBCm was 7 to 8 nm, the increase in particle size of RBCm-LCNPs was attributed to the lipid bilayer of RBCm. After modification with HA-conjugated dioleoyl phosphoethanolamine (HA-DOPE), the particle size increased to 220 nm (Additional file 1: Figure S1A). The increase in the negative charge of RBCm-LCNPs relative to non-coated LCNPs improved nanoparticle stability in the blood [28, 29]. LCNPs appeared round, showed uniform morphology and distribution, and were non-adhesive. After the cell membrane was coated, a clear core-membrane structure was observed (Fig. 2B). The LCNPs had a honeycomb or sponge-like internal structure, which showed no significant changes after cell membrane coating (Fig. 2C). The drug-loading and encapsulation efficiencies of the nanocarriers were 8.7 and 95% for PTX, respectively, and 1.7 and 93% for IR780, respectively. Thus, the multi-chambered structure of LCNP we produced had excellent drug-loading.

The prolonged circulation of RBCs in the blood is mediated by specific membrane proteins [30]. Most RBCm proteins, including CD47, were retained on RBCm-LCNPs and HA&RBCm-coated LCNPs (HA&RBCm-LCNPs) (Fig. 2D). CD47 is a self-marker protein that actively signals macrophages [31]. The signal-regulated protein alpha glycoprotein expressed by phagocytic cells, recognizes CD47 as a “don’t eat me” signal to prevent immune cells from inducing RBC phagocytosis [32, 33].

No significant change in particle size of LCNPs and RBCm-coated nanoparticles was found in the plasma for 72 h (Additional file 1: Figure S1B), which suggests potential for long circulation of the nanocarriers in the blood. No precipitation and aggregation were found in the blood, and there was no hemolysis in the range of 0.5–50 µg/mL (PTX) for RBCm-LCNPs and HA&RBCm-LCNPs; in contrast, precipitation, aggregation, and hemolysis were observed in the saline group (Additional file 1: Figure S1C). These findings imply that the blood compatibility of LCNPs were improved by RBCm and HA&RBCm coating; therefore, these systems were deemed suitable for intravenous administration. The cumulative in vitro release of both PTX and IR780 from LCNPs for 72 h were about 80%, and both systems exhibited synchronous release. After being coated with RBCm and being modified with HA, the degree of sustained release was more significant (Additional file 1: Figure S2). Sustained release has been reported to be associated with the prolonged circulation time of the loaded hydrophobic drug in the blood [34].

**Fig. 3:**
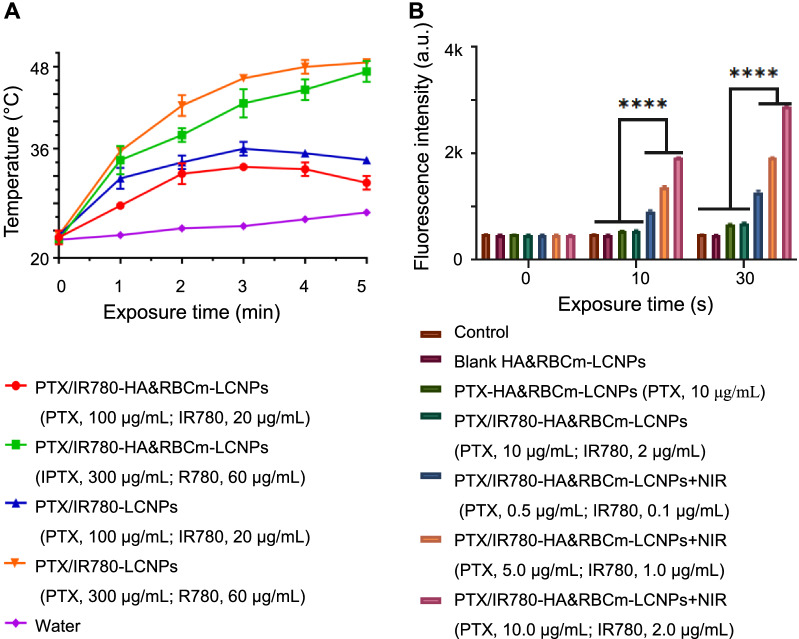
Photothermal and photodynamic characteristics of PTX/IR780-loaded nanocarriers. **A** heating profiles of the water and nano-formulation solutions following near-infrared irradiation (NIR: 808 nm, 1 W/cm^2^). **B** the fluorescence intensity of SOSG in water (Control) and nano-formulation solutions (n = 3). ****p < 0.0001

The maximum absorption wavelength of IR780 in the nanoparticle group was slightly higher (red shift) than that of IR780 in solution (Additional file 1: Figure S3A), which may be induced by the increase of internal stress, decrease of energy gap, and vacancy effect caused by the nanocarriers, suggesting that IR780 was successfully encapsulated in LCNPs. IR780 quenching was faster when the particles were exposed to normal light than when protected from light, and quenching slowed down following encapsulation by nanoparticles. HA&RBCm-LCNPs showed better protection of IR780 than LCNPs (Additional file 1: Figure S3B–D). When irradiated with near-infrared (NIR) light (808 nm), the quenching of IR780 in the HA&RBCm-LCNP group was slower than that in the LCNP group, but IR780 in both groups was completely quenched 5 min post-exposure (Additional file 1: Figure S3E). Therefore, the NIR exposure time was set to 5 min in subsequent phototherapy experiments. The IR780-loaded nanocarriers in aqueous solution exhibited a gradual dose-dependent increase in temperature following NIR irradiation (Fig. 3A). At an IR780 concentration of 60 µg/mL, the water temperature increased to above 45 °C within 5 min, but at low concentration (20 µg/mL), the water temperature did not exceed 37 °C. Although it was difficult to achieve photothermal effects at low concentrations, we found that even at very low concentrations of IR780 (0.1 µg/mL), the fluorescence intensity of Singlet Oxygen Sensor Green® (SOSG) generated after NIR irradiation for only 10 s was much greater than those of the control (water) and non-NIR-treated groups (*p* < 0.0001), suggesting that IR780 induced the production of a large amount of singlet oxygen, and the yield grew over a prolonged duration and increased IR780 concentration (Fig. 3B). These results demonstrate that PTX/IR780-HA&RBCm-LCNPs have excellent photodynamic effects.

### In vitro cellular uptake

**Fig. 4:**
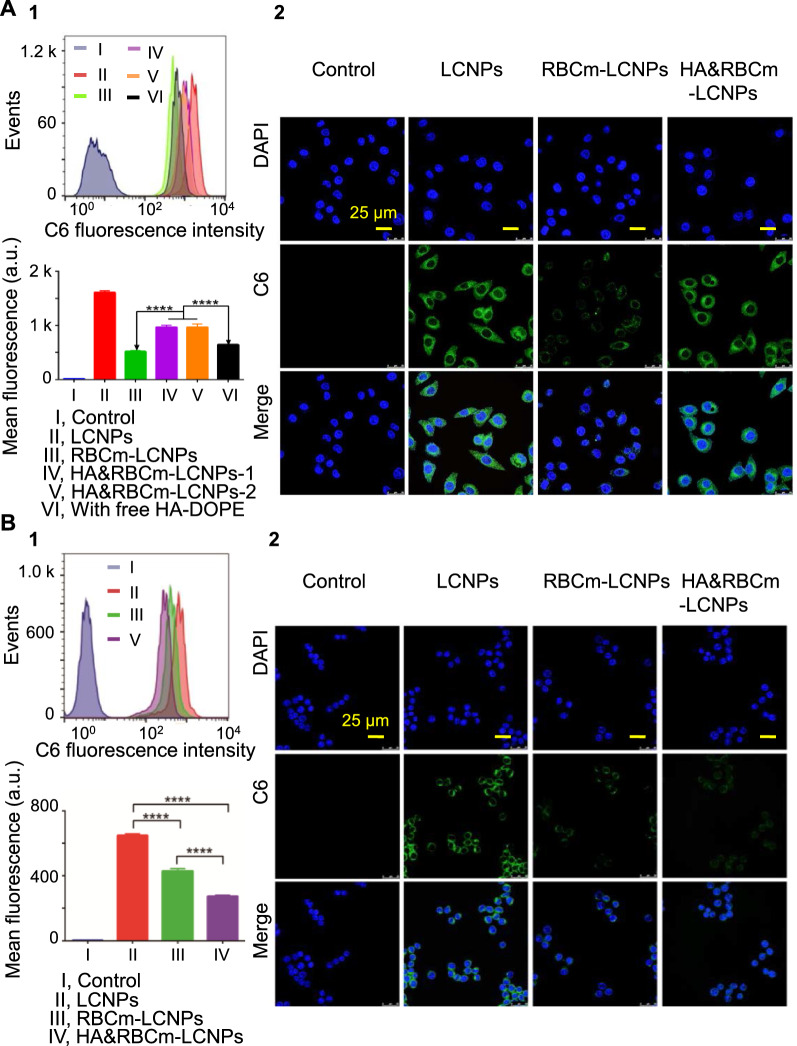
Uptake of C6-labeled nanocarriers by **A** A549 and **B** RAW264.7 cells. HA&RBCm-LCNPs-1 and -2 refer to LCNPs with HA-DOPE concentrations of 0.5 and 1.0 mg/mL, respectively. The “Control” group represents untreated cells; the “with free HA-DOPE” group was administered HA&RBCm-LCNPs without the removal of free HA-DOPE during the final process. Analysis was performed through flow cytometry (1; top, FACS analysis of the C6 signal; bottom, statistical analysis of differences in the fluorescence intensity among the groups) and confocal laser scanning microscopy (2; nucleus was labeled with DAPI) (n = 3). ****p < 0.0001

To determine the tumor accumulation potential of nanocarriers and their stealth properties in the blood, tumor cells (A549) with high expression levels of CD44 as a primary receptor for the macromolecule HA, and macrophages with RES phagocytic function (RAW264.7) were observed for their ability to take up the nanocarriers involved in this study. Although the uptake of IR780 and paclitaxel by cells was not measured, the ability of different nanocarriers to be internalized by cells was successfully evaluated using C6 as a fluorescent probe. Even at a very low final concentration of C6 in the medium (1 µg/mL), the C6 fluorescence in the cells was very strong after being incubated with C6-encapsulated nanocarriers for 2 h, resulting in the fluorescence-activated cell sorting (FACS) signal peaks were not completely separated (Fig. 4A), but the statistical results of the fluorescence intensity of each test group showed that there were extremely significant differences between different nanocarriers. The fluorescence intensity of A549 cells in the RBCm-LCNP group was lower than that in the LCNP group, which could be attributed to the increased interaction between the nanoparticles and the cells, owing to the increased negative charge on the surface of RBCm-coated LCNPs. Following HA modification, cellular uptake of nanoparticles substantially increased. The fluorescence intensity in the HA&RBCm-LCNP (HA-DOPE: 0.5 mg/mL) group was 1.87-fold higher than that in the RBCm-LCNP group. However, cellular uptake of the RBCm-LCNPs modified with different HA-DOPE concentrations (0.5 mg/mL and 1.0 mg/mL) at the point where CD44 was saturated with HA did not significantly differ (*p* > 0.05). Moreover, the fluorescence intensity in the HA&RBCm-LCNP group with free HA-DOPE was markedly lower than that in the two HA&RBCm-LCNP groups with no free HA-DOPE competitively bound to the CD44 receptor of A549 cells (*p* < 0.0001). Considering that using a large number of cells may yield false negative results in FACS, the nanocarriers internalized by cells were further visualized via confocal laser scanning microscopy (CLSM). Post-incubation with the coumarin 6 (C6)-labeled preparation showed that green fluorescence of C6 was primarily distributed in the cell membrane and the cytoplasm, and no signal was observed in the 4ʹ,6-diamidino-2-phenylindole (DAPI)-labeled nucleus with blue fluorescence (Fig. 4A). CLSM imaging also showed that the fluorescence intensity in the RBCm-LCNP group was remarkably lower than that in the LCNP group, whereas that in the HA&RBCm-LCNP group was markedly higher. These results correspond with the results of the flow cytometric analysis and confirmed that HA&RBCm-LCNP actively target A549 cells.

Escaping phagocytosis by macrophages is a key factor for the extended circulation of nanoparticles in the blood. RBCm-coated nanoparticles reduced the rate of macrophage phagocytosis, as shown by a notable decrease in the RAW264.7 cell uptake of RBCm-coated nanoparticles compared to that of LCNPs without RBCm coating (*p* < 0.0001) (Fig. 4B). Furthermore, HA modification masked the RBCm-uncoated regions of the nanoparticles, improved the sustained release of the liposoluble cargo (C6), which resulted in a highly significant reduction in the uptake of HA&RBCm-LCNPs [35]. After ingestion by RAW264.7 cells, C6 was primarily distributed in the cell membrane and cytoplasm and did not accumulate in the nucleus. The weakest fluorescence signals detected through CLSM were observed in the HA&RBCm-LCNP group. The successful transfer of RBCm proteins, such as CD47, to the LCNPs is key for weakening the immune response by presenting drug-loaded nanocarriers as “self,” ultimately leading to long-term circulation of LCNPs in the blood [36, 37].

### In vitro antitumor effects

Photochemotherapy produced by the combination of PTX and IR780 has been shown to enhance antitumor effects [38]. For example, the cure rate of primary melanoma-bearing mice with intratumoral injection was 100% within 30 days [39]; IR780 was able to exert photothermal therapeutic effects on pancreatic ductal adenocarcinoma and expanded the tumor interstitial space to promote paclitaxel delivery to deep tumor tissues [40]. However, IR780 is a NIR-I near-infrared photosensitizer with a limited tissue-penetrating depth [41]. As a result, photothermal therapy administered via intravenous injection generally requires a higher dose than local administration (for mice, the intravenous dose generally exceeds 1 mg/kg) (Additional file 1: Table S1) and requires coupling with high-intensity near-infrared irradiation to achieve the required temperature. Thus, the precise control of the required temperature and the gradual attenuation of light intensity for tissue penetration remains challenging. Failure to control temperature and light intensity often lead to tissue perforation with local overheating, and failure in thermal ablation of large-volume tumors; therefore, the type I near-infrared photosensitizer-mediated photothermal therapy including IR780 has potential mainly in the treatment of superficial solid tumors [42, 43]. Low-dose photodynamic therapy (PDT) has been reported to induce the generation of endogenous reactive oxygen species (ROS) in the electron transport chain of mitochondria, and additional ROS promoted apoptosis [44]. Due to the ability of IR780 to target mitochondria, PDT can be achieved at a lower dose than that used for regular photothermal therapy [45]. Therefore, we focused on IR780-mediated PDT in combination with PTX treatment, with the eventual aim of improving antitumor therapy and reducing side effects.

The anti-tumor effects of functionalized nanoparticles are usually related to their long-term circulation, targeting, controlled release and other behaviors in vivo. However, the in vivo microenvironment is difficult to be simulated in an in vitro cell culture model. Therefore, we only investigated the effect of HA&RBCm-LCNPs-mediated photochemotherapy on A549 cells cultured in vitro, but did not compare the actions of different nanocarriers. Blank HA&RBCm-LCNPs had little effect on cell viability (Additional file 1: Figure S4). The cytotoxicity of each preparation increased in a concentration-dependent manner within the ranges of 0.05–20 µg/mL paclitaxel and 0.01–4 µg/mL IR780. The NIR-treated PTX/IR780-loaded groups achieved higher levels of cytotoxicity than the groups without NIR (*p* < 0.01), indicating that the combination of chemotherapy and phototherapy using PTX and IR780 was better than chemotherapy using PTX alone. Within the NIR-treated groups, the IC_50_ in the HA&RBCm-LCNP group was lower than that in the RBCm-LCNP group (Additional file 1: Table S2), owing to the receptor (CD44) and ligand (HA) interaction that significantly increased nanoparticle uptake by A549 cells [46, 47]. In addition, the IC_50_ value in the NIR-treated PTX-HA&RBCm-LCNP group was slightly lower than that in the non-NIR-treated PTX/IR780-HA&RBCm-LCNP group (1.84 vs. 2.00), owing to the low dark toxicity of IR780 in the concentration range examined.

**Fig. 5:**
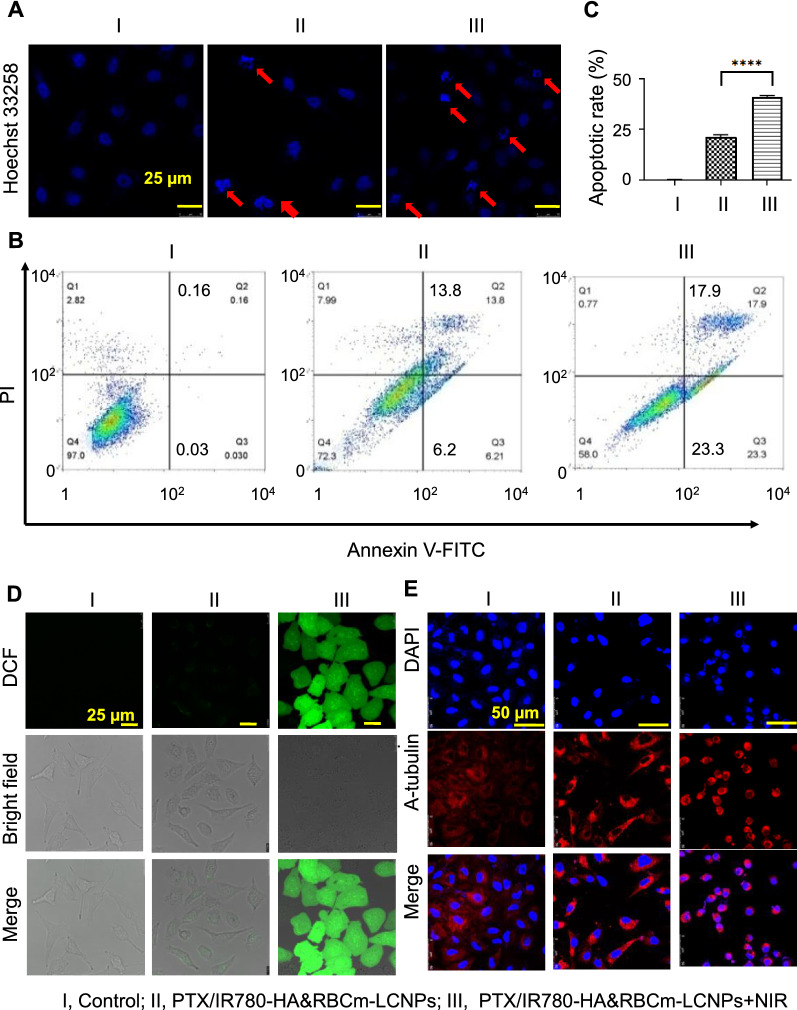
Apoptosis of A549 cells in vitro following treatment with PTX and IR780 co-loaded preparations. The “Control” (I), “PTX/IR780-HA&RBCm-LCNPs” (II), and “PTX/IR780-HA&RBCm-LCNPs + NIR” (III) groups refer to cells not treated with drugs, non-NIR treated nano-formulations, and NIR-treated nano-formulations, respectively. **A**, **D**, **E** show confocal laser scanning microscope images of cells with Hoechst 33,258-labeled nuclei, 2′,7′-Dichlorodihydrofluorescein diacetate (DCFH-DA)-labeled reactive oxygen species, and microtubules, respectively. **B**, **C**, Apoptosis measured via flow cytometry. For the phototherapy group, cells were exposed to NIR at 808 nm (1 W/cm^2^) for 5 min 2 h post-drug administration. The final PTX and IR780 concentrations were 5 µg/mL and 1 µg/mL, respectively (n = 3). ****p < 0.0001

Photochemotherapy conspicuously increased apoptosis compared with chemotherapy (Fig. 5A), which was confirmed via flow cytometry. The rate of A549 apoptosis was higher in the NIR-treated PTX/IR780-HA&RBCm-LCNP group than in the control and non-NIR-treated PTX/IR780-HA&RBCm-LCNP groups (*p* < 0.0001; Fig. 5B, C).

Previous reports have shown that the enhanced outcomes of combinational treatment with PTX and IR780 were associated with the promotion of tumor cell apoptosis via a prolonged G2 phase of the cell cycle and ROS-mediated PDT, which were in agreement with our findings [38, 39]. Following exposure to NIR, PTX/IR780-HA&RBCm-LCNPs successfully induced A549 cells to produce a large amount of ROS, whereas without NIR irradiation, no obvious ROS production was observed (Fig. 5D). Tubulin is the target through which PTX inhibits tumor cell proliferation. PTX binds tubulin to prevent the compression of α- and β-tubulin, resulting in no tension accumulation and depolymerization [48]. The microtubules in the cells in the control group were morphologically regular, densely distributed in the cytoplasm, clearly filamentous, and formed a fusiform network-like structure around the nuclei (Fig. 5E). The cell morphology in the non-NIR-treated PTX/IR780-HA&RBCm-LCNP group did not change conspicuously but the number of cells was reduced, microtubules gathered around the nuclei, and the microtubules were irregularly shape with unclear structure, which resulted in a loss of cellular polarity. In the NIR-treated PTX/IR780-HA&RBCm-LCNP group, the cells shrunk significantly and were further reduced in number, with a diminution in microtubules and an obvious change in morphology, indicating that photochemotherapy inhibited cell proliferation and the production and depolymerization of microtubules.

**Fig. 6:**
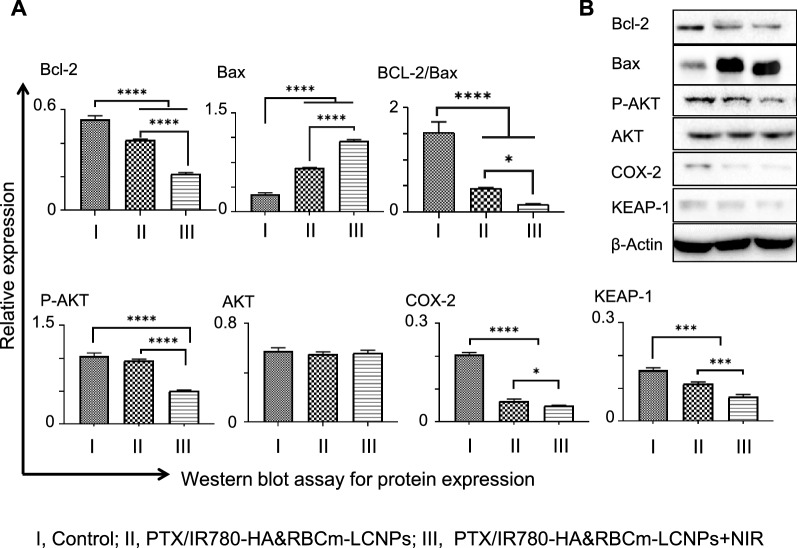
Changes in the expression of apoptosis-associated proteins in A549 cells treated with PTX and IR780 co-loaded preparations. The “Control” (I), “PTX/IR780-HA&RBCm-LCNPs” (II), and “PTX/IR780-HA&RBCm-LCNPs + NIR” (III) groups refer to cells not treated with drugs, non-NIR treated nano-formulations, and NIR-treated nano-formulations, respectively. **A** the apoptosis-related proteins assayed through western blotting (n = 3); **B** western blotting bands. For the phototherapy group, cells were exposed to NIR at 808 nm (1 W/cm^2^) for 5 min 2 h post-drug administration. *Bcl-2* B-cell lymphoma 2, *Bax* Bcl-2-associated X protein, *COX-2* cyclooxygenase-2. ****p < 0.0001, ***p < 0.001, *p < 0.05

We further found that the NIR-treated PTX/IR780-HA&RBCm-LCNPs promoted apoptosis not only by destroying the microtubules but also by stimulating apoptosis-related proteins, such as the PDT-induced endogenous mitochondrial apoptotic pathway resulting from the preferential accumulation of IR780 in the mitochondria of tumor cells, which was associated with B-cell lymphoma 2 (Bcl-2) and Bcl-2-associated X protein (Bax) (Fig. 6A, B) [45, 49]. Bcl-2 is an anti-apoptotic protein that maintains the mitochondrial membrane potential and blocks the release of cytochrome C [50]. Bax is a pro-apoptotic protein that eliminates the mitochondrial membrane potential to promote the release of cytochrome C, thereby activating the enzyme caspase cascade, and ultimately leading to apoptosis. Bcl-2 and Bax in the cells in the drug-treatment groups were down- and upregulated, respectively (*p* < 0.0001), relative to the control group. The ratios of Bcl-2 to Bax (Bcl-2/Bax) in the drug-treatment groups were remarkably decreased relative to the control group (*p* < 0.0001), indicating that PTX/IR780-HA&RBCm-LCNP induced apoptosis through the endogenous mitochondrial pathway. Notably, the NIR-treated group showed stronger regulatory effects on Bcl-2 and Bax than the non-NIR-treated PTX/IR780-HA&RBCm-LCNP group (*p* < 0.0001), as shown by a lower Bcl-2/Bax ratio (*p* < 0.05), suggesting that ROS-induced apoptosis was involved.

Serine/threonine kinase Akt, another proto-oncogene involved in the intracellular conduction pathway, is activated by phosphatidylinositol 3-kinase (PI3K) to generate phosphorylated-Akt (p-Akt), inhibit apoptosis, and promote cell cycle progression [51]. In our study, the PTX/IR780-HA&RBCm-LCNPs with or without NIR-treatment had no noticeable effects on the total Akt expression levels in A549 cells (*p* > 0.05). However, NIR treatment of PDT inhibited the activation of the PI3K/Akt pathway by downregulating p-Akt, relative to the control and non-NIR-treated PTX/IR780-HA&RBCm-LCNP groups (*p* < 0.0001), thereby sensitizing cells to ROS-mediated apoptosis [52, 53]. Furthermore, Kelch-like ECH-associated protein 1 (Keap1), the main inhibitor of the nuclear factor erythroid 2-related factor 2 (Nrf2), was also significantly inhibited in A549 cells in the treated groups relative to the control group (*p* < 0.001), and the expression levels of Keap1 in the NIR-treated group was lower than that in the non-NIR-treated group (*p* < 0.001). Keap1/Nrf2 is an important pathway for anti-oxidative responses in cells [54]. PDT produces excess ROS and downregulates Keap1, thereby triggering apoptosis; the PI3K/Akt pathway initiates the production of ROS by weakening Nrf2 stability, which contributes to sensitization and the reversion of resistance to chemotherapeutic drugs such as PTX [55, 56]. Therefore, we speculated that the effects of NIR-treated PTX/IR780-HA&RBCm-LCNPs on p-Akt and Keap1 has the potential to reverse the resistance of A549 cells to PTX. We also found that PTX/IR780-HA&RBCm-LCNPs substantially lowered the expression of intracellular cyclooxygenase-2 (COX-2) (*p* < 0.0001), and NIR irradiation probably enhanced this downregulation (*p* < 0.05). COX-2 expression has been reported to upregulate Bcl-2 in rat intestinal epithelial cells, repress the cleavage of caspase-3 in oral squamous cell carcinoma, and is accompanied by the increased phosphorylation of Akt in endometrial cancer cells, thereby inhibiting tumor cell apoptosis and promoting tumor growth [57–59]. It also upregulates membrane-type matrix metalloproteinases (MMPs), such as MMP-2, in human colon cancer cells to promote tumor cell infiltration and metastasis [60]. Therefore, the inhibition of COX-2 can reverse the inhibition of tumor cell apoptosis and migration. However, a previous report has shown that hypericin-mediated PDT increases the amount of COX-2 in HeLa cells and a T24 (human transitional cell carcinoma of the urinary bladder) cell culture [61]. It can be inferred that the regulatory effects of PTX/IR780-HA&RBCm-LCNPs on COX-2 in A549 cells is related to the inhibition of COX-2 expression by PTX and IR780 [62].

**Fig. 7:**
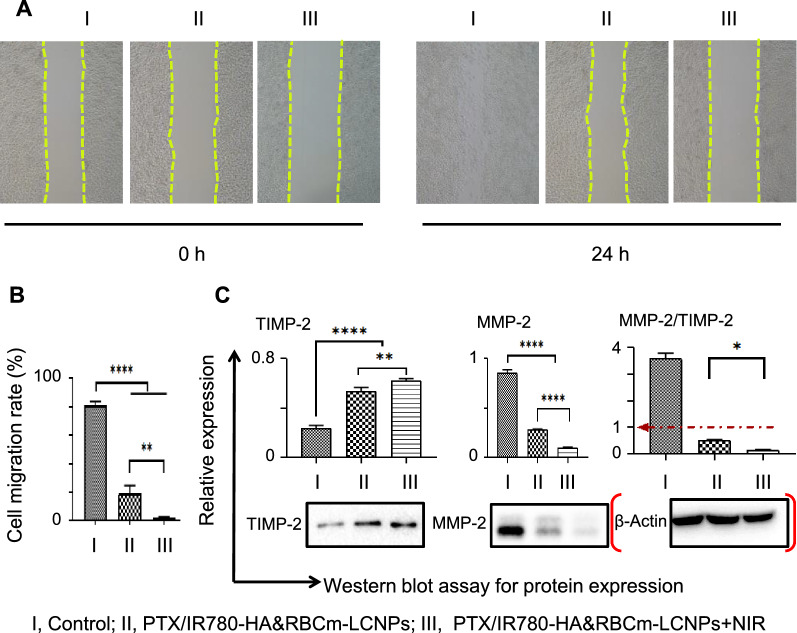
Migration of A549 cells in vitro following treatment with PTX and IR780 co-loaded preparations. The “Control” (I), “PTX/IR780-HA&RBCm-LCNPs” (II), and “PTX/IR780-HA&RBCm-LCNPs + NIR” (III) refer to cells not treated with drugs, non-NIR treated nano-formulations, and NIR-treated nano-formulations, respectively. **A** Cell migration micrographs; **B** cell migration rates; **C** related proteins assayed by western blotting. The final PTX and IR780 concentrations were 2.5 µg/mL and 0.5 µg/mL, respectively (n = 3). For the phototherapy group, cells were exposed to NIR at 808 nm (1 W/cm^2^) for 5 min 2 h post-drug administration. *MMP-2* matrix metalloproteinase 2; tissue inhibitor 2 of matrix metalloproteinase-2, TIMP-2. ****p < 0.0001, **p < 0.01, *p < 0.05

The ability to PTX/IR780-HA&RBCm-LCNPs to disrupt microtubules and to regulate migration-associated signals, including the aforementioned protein pathways, lead to the reduced migratory capacity of A549 cells [60, 63]. Because the drug concentration that was effective in the apoptosis experiment had too much inhibitory effects on cell migration, the effects of the different treatment groups on migration were difficult to assess. Thus, we reduced the concentrations of PTX and IR780 to 2.5 µg/mL and 0.5 µg/mL, respectively. Micrographs of the changes in scratches at 0 and 24 h are shown in Fig. 7A. At 24 h, the cell migration rate was 91.81% in the control group, and almost all scratches had healed. However, the cell migration rate in each nano-formulation-treated group was significantly less than that in the control group (Fig. 7B; *p* < 0.0001). Moreover, the migration rate in the NIR-treated PTX/IR780-HA&RBCm-LCNP group was significantly lower than that in the non-NIR-treated group (*p* < 0.01). The results corresponded with the expression levels of MMP-2 and tissue inhibitor 2 of matrix metalloproteinase-2 (TIMP-2) in the tested groups (Fig. 7C). High expression levels of MMP-2 is closely related to infiltration and vascular invasion of the basement membrane and the interstitium as well as to the metastasis of tumor cells. However, MMP-2 is inhibited by TIMP-2 [60]. In our study, the expression of these two proteins were dramatically inhibited in A549 cells in the treated groups (*p* < 0.0001); the NIR-treated PTX/IR780-HA&RBCm-LCNPs downregulated MMP-2 (*p* < 0.0001) and TIMP-2 (*p* < 0.01), and the MMP-2/TIMP-2 ratio (less than 1 in both administration groups) was lower than that in the group without NIR irradiation (*p* < 0.05), suggesting that NIR-treated PTX/IR780-HA&RBCm-LCNPs induced A549 apoptosis and inhibited migration via microtubule-, ROS-, and mitochondrial stress-related pathways. The inhibition of migration also likely involved the MMP-2/TIMP-2 signaling pathway and COX-2 regulation.

### Circulation and tumor targeting capacity in vivo

**Fig. 8:**
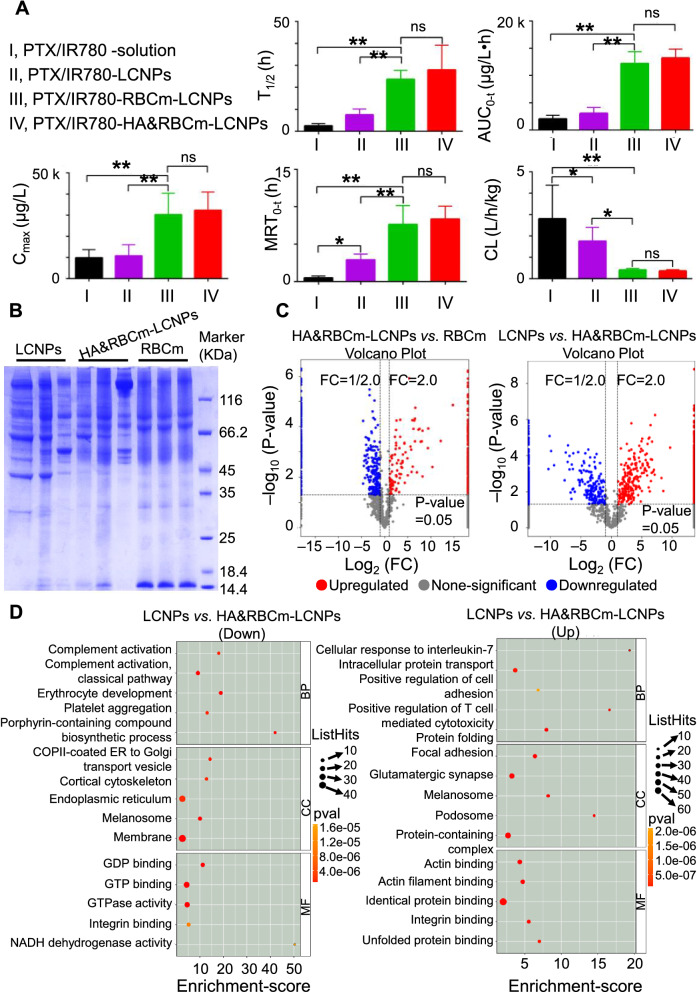
Circulation of nanoparticles in vivo. **A** In vivo pharmacokinetic properties of PTX, and the biodistribution of PTX and IR780 following intravenous administration of PTX and IR780 co-loaded preparations in rats. The doses of PTX and IR780 were 5 mg/kg and 1 mg/kg, respectively (n = 6). **B** Results of crown protein analysis via sodium dodecyl sulfate-polyacrylamide gel electrophoresis. **C** Bubble map of the top 15 entries from a Gene Ontology (GO) enrichment analysis. The GO entries were screened for differential proteins with scores greater than 1 in the three categories. Then, the top 5 corresponding −log_10_ p-values were obtained from each entry. Entries with larger bubbles indicate more proteins. Color change from yellow to red suggests that the enrichment p-value is smaller and the significant difference is greater. NS, not significant; *p* > 0.05; *p < 0.05, **p < 0.01

Our in vitro uptake experiments showed that HA&RBCm-LCNPs effectively decreased macrophage phagocytosis, which may lead to extended circulation in the blood. This was confirmed by the subsequent in vivo pharmacokinetic studies in rats (Fig. 8A). Following intravenous administration of the formulations, the plasma concentrations of PTX in the PTX/IR780-solution group and the PTX/IR780-LCNP group were less than the detection limit at 8 and 24 h, respectively. In contrast, the PTX/IR780-RBCm-LCNPs and PTX/IR780-HA&RBCm-LCNPs were detectable up to 72 h post-administration (Additional file 1: Figure S5). The half-life (T_1/2_), area under the curve (AUC_0−t_), and peak concentration (C_max_) in the PTX/IR780-RBCm-LCNP group were 9.56- and 3.14-fold, 5.91- and 3.97-fold higher than in the PTX/IR780-solution group, respectively, and were 3.08- and 3.26-fold higher than in the PTX/IR780-LCNP group, respectively. The mean residence time (MRT_0 − t_) in the PTX/IR780-solution group was only 0.5 ± 0.25 h, whereas those in the PTX/IR780-LCNP and PTX/IR780-RBCm-LCNP groups were 5.8- and 15.24-fold higher, respectively. The clearance rate in the PTX/IR780-RBCm-LCNP group was also significantly lower than those in the PTX/IR780-solution and PTX/IR780-LCNP groups.

The RBCm-camouflaged LCNPs could successfully escape from phagocytosis by macrophages, effectively prolonging the t_1/2_ and MRT_0−t_ of PTX, and marginally improving drug bioavailability [64]. A previous study has shown that RBCm-coated nanoparticles have a t_1/2_ of 39.6 h, which is more than twice the t_1/2_ of PEGylated nanoparticles (15.8 h) [65]. Chao et al. reported that, compared with non-PEGylated liposomes, PEGylated liposomes adsorbed less proteins in the blood, which mainly reduced the affinity of immunoglobulins. However, apolipoprotein E, which has been shown to retard the phagocytosis of macrophages, was not found in the protein corona of PEGylated liposomes [66]. In our work, the protein compositions in the RBCm and the blood-treated nanocarriers (LCNPs and HA&RBCm-LCNPs) were quite different (Fig. 8B, C). The amounts of fibrinogen gamma chain (50.632 kDa), an important protein in the protein crown, in the LCNP and HA&RBCm-LCNP groups were not statistically different (*p* > 0.05) (Additional file 2) [67]. Immunoglobulins and complement proteins are generally considered to induce opsonization and RES recognition [68, 69]. The amounts of immunoglobulins (Igs), such as Ig gamma-2 A chain C region (35.18 kDa), Ig gamma-2B chain C region (36.49 kDa), and Ig gamma-2 chain C region (11.37 kDa) in the protein crowns of LCNPs and HA&RBCm-LCNPs were also not statistically different (*p* > 0.05). By contrast, certain complement proteins, such as C3 (186.32 kDa), C4 (192.16 kDa), C5 (189.08 kDa), C9 (62.28 kDa), C1q subcomponent subunits-A (25.917 kDa), -B (26.647 kDa) and -C (25.686 kDa), which may not shorten the circulation time of nanocarriers, were higher in the HA&RBCm-LCNP group than in the LCNP group (Fig. 8D; Additional file 2). However, the amount of leukocyte surface antigen CD47 (32.995 kDa) in the HA&RBCm-LCNP group contained was significantly higher than that in the LCNP group (*p* < 0.05). Meanwhile, the amounts of certain myosins, including tropomyosin alpha-3 chain (29.006 kDa), tropomyosin alpha-4 chain (28.509 kDa), and filamin A (280.49 kDa), that adhered to the LCNPs were markedly higher than those that adhered to the HA&RBCm-LCNPs (*p* < 0.05). CD47 protein is recognized as a “Don’t eat me” signal protein, and myosin, such as myosin VII, has been reported to be positively related to phagocytosis by macrophages [70]. In conclusion, the prolonged circulation of HA&RBCm-LCNPs in the blood is considered to have been achieved via the following: (1) alterations to the types rather than the amounts of proteins attached to the surface of the nanoparticles; (2) an abundance of CD47 and reduced affinity to myosins; and (3) excellent sustained-release properties. In addition, apolipoprotein E was detected in both LCNP and HA&RBCm-LCNP groups (*p* > 0.05), suggesting that the circulatory properties of the tested nanocarriers are distinct from those of PEGylated nanoparticles [66].

The pharmacokinetic parameters in the PTX/IR780-HA&RBCm-LCNP group did not statistically differ from those in the PTX/IR780-RBCm-LCNP group, indicating that HA modification had no apparent effects on the circulation of PTX/IR780-RBCm-LCNPs. However, although not statistically different, the circulation of HA&RBCm-LCNPs was superior to those of RBCm-LCNPs as suggested by the parameters, which partly echoes the results of the macrophage phagocytosis studies in vitro and also demonstrates the more complex fate of nanocarriers in vivo.

**Fig. 9:**
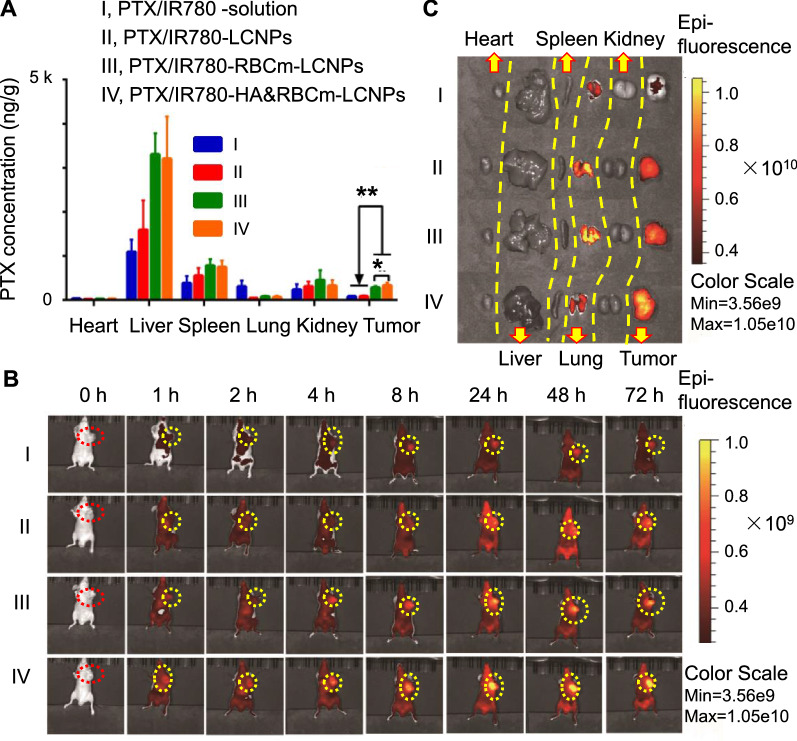
In vivo biodistribution of **A** PTX and **B**, **C** IR780 in tumor-bearing nude mice following intravenous administration of PTX and IR780 co-loaded preparations for 6 h (PTX) and over 72 h (IR780). The doses of PTX and IR780 were 5 mg/kg and 1 mg/kg, respectively (n = 6). NS, not significant; *p* > 0.05; *p < 0.05, **p < 0.01

HA&RBCm-LCNPs possess multiple characteristics, including sustained release, long circulation in the blood, and active targeting, that contribute to increased accumulation in tumors. Six hours following administration, PTX accumulation in the tumor in the PTX/IR780-HA&RBCm-LCNP group was 4.33- and 4.23-fold higher than that in the PTX/IR780-LCNP group and the PTX/IR780-solution group, respectively, and was significantly higher than that in the PTX/IR780-RBCm-LCNP group (*p* < 0.05) (Fig. 9A). PTX accumulation was lowest in the heart of tumor-bearing nude mice. However, PTX accumulation in the liver and spleen of mice in the RBCm-coated group was higher than in mice in the LCNP group. This is probably due to the fact that, as reported, the rich blood stored in these two isolated tissues was not removed through perfusion before sample preparation, and the nanoparticles in the blood contributed a large amount of drugs, further demonstrating the excellent circulation profile of the RBCm-coated nanocarriers [71].

The dynamic tissue distribution of nano-formulations in tumor-bearing nude mice was visually monitored using small animal imaging systems. IR780 has the property of targeting the mitochondria and allows long-term imaging in vivo. Previous reports have shown that mice had strong in vivo fluorescence signals until 48 h after intravenous administration of free IR780; Yang et al. and Zhang et al. demonstrated that at 48 h after administration of IR780-loaded nanoparticles, the fluorescence intensity at the tumor site was not significantly different or stronger than that at 12 h after administration [72–74]. Here, we found that after 72 h of intravenous administration, the mice in each group still exhibited IR780 fluorescence, but the fluorescence intensity was remarkably reduced. Since the drug in the systemic circulation gradually decreased over time, the drug distribution in the isolated organs at 72 h had noticeable differences from those observed at 6 h after administration. The fluorescence of IR780 at the tumor site of the PTX/IR780-HA&RBCm-LCNP group was the highest among the tested groups, and the fluorescence intensity increased with time (Fig. 9B). Fluorescence in the lungs and tumors of mice in the PTX/IR780-LCNP and PTX/IR780-RBCm-LCNP groups were higher than those in the PTX/IR780-solution group. In the PTX/IR780-HA&RBCm-LCNP group, the fluorescence intensity in the tumor was further increased, while that in the lung was reduced likely owing to the HA-mediated tumor-targeting capacity of the nanocarriers.

### In vivo antitumor activity

**Fig. 10:**
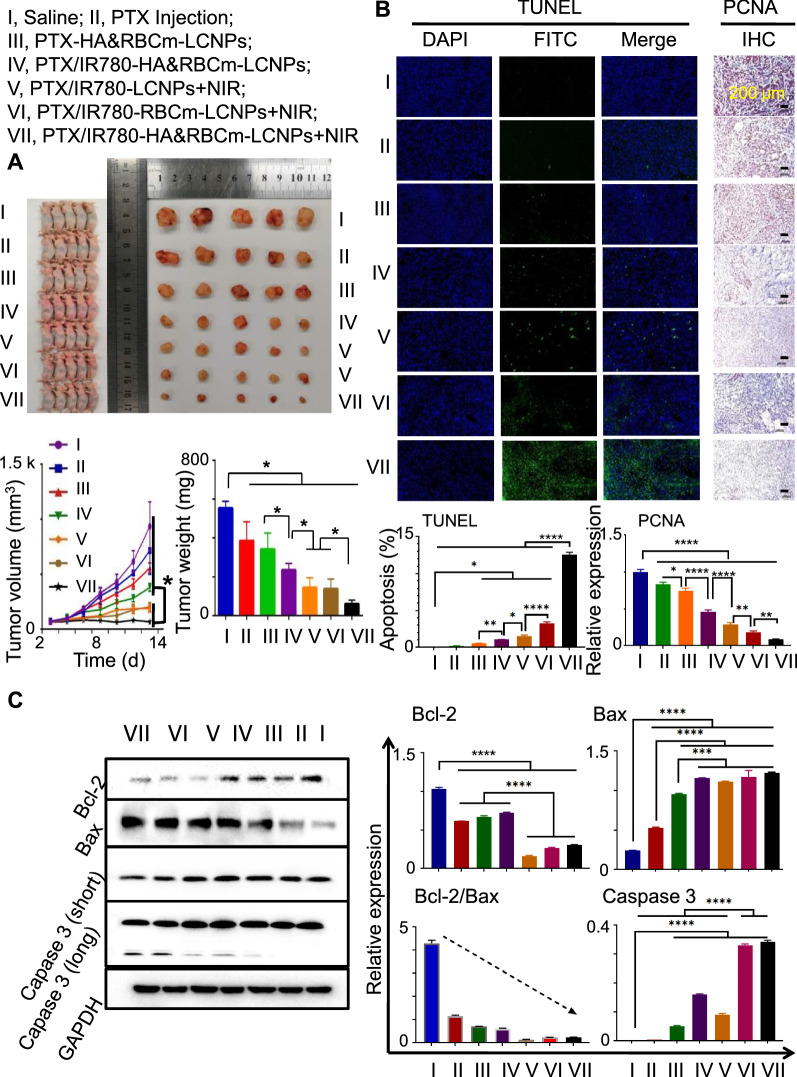
In vivo antitumor effects of normal saline and drug-loaded preparations intravenously administered to tumor-bearing BALB/c nude mice once every 2 days for 12 days. Doses: PTX, 5 mg/kg; IR780, 1 mg/kg. Normal saline: Saline, control. **A** Images of tumors, tumor volume changes, and tumor weights at the end of the 13-day treatment period. **B** Micrographs of tumor tissue slices for proliferating cell nuclear antigen (PCNA) and terminal deoxynucleotidyl transferase dUTP nick-end labeling (TUNEL) analysis.** C** Western blotting for apoptosis-related proteins (n = 5). For the phototherapy group, animals were exposed to NIR at 808 nm (1 W/cm^2^) for 5 min 2 h post-drug administration. ****p < 0.0001, ***p < 0.001, **p < 0.01, *p < 0.05

During the 13-day treatment period, the tumor volume increased in varying degrees in each group of tumor-bearing nude micetumor-bearing nude mice. The most pronounced increase was observed in the normal saline group and the commercial PTX Injection (normal aqueous solution) group (Fig. 10A). During the experimental duration, the increase in tumor volume in the NIR-treated groups were slower than those in the non-NIR-treated groups (*p* < 0.05). In particular, tumor volume in the NIR-treated PTX/IR780-HA&RBCm-LCNP group was maintained at ~ 200 mm^3^ and was the smallest among all groups following treatment (*p* < 0.05). At the end of treatment, the drug administration groups achieved antitumor efficacy, with tumor weights lower than those in the saline control group (*p* < 0.05). Among the NIR-treated groups, the tumor weight in the PTX/IR780-HA&RBCm-LCNP group was the lowest (*p* < 0.05). These results demonstrate that RBCm coating prolonged the circulation of nanoparticles and improved the antitumor effects of drug-loaded nanocarriers, and these effects were further enhanced by HA modification [75]. Furthermore, the antitumor effects of combined chemotherapy and PDT produced by PTX and low-dose IR780 was superior to the effects of chemotherapy alone, as the tumor weights in NIR-treated groups was lower than those in non-NIR-treated groups (*p* < 0.05).

The exposed 3ʹ-OH in fragmented genomic DNA in apoptotic cells was labeled with fluorescein isothiocyanate (FITC) through the terminal deoxynucleotidyl transferase dUTP nick-end labeling (TUNEL) method, and tumor proliferation was evaluated through the detection of proliferating cell nuclear antigen (PCNA). The microscopy images (Fig. 10B) showed that the saline group had no obvious fluorescent markers in the tumor tissues, whereas the PTX Injection and non-NIR-treated PTX/IR780-HA&RBCm-LCNP groups had only sporadic fluorescent markers, showing a few apoptotic cells. In contrast, there was dramatically more scattered TUNEL-stained green fluorescence in the NIR-treated groups (*p* < 0.0001). The number of fluorescent-positive cells was higher in the RBCm-coated groups than that in the uncoated nanoparticle group (*p* < 0.0001); the PTX/IR780-HA&RBCm-LCNP group had the greatest fluorescence distribution (*p* < 0.0001), indicating the highest number of apoptotic cells. The results of the PCNA analysis were similar to those of the TUNEL assay. PCNA-positive staining was primarily observed in the nuclei and was indicated by brown spots (Fig. 10C). The PCNA-positive to total cell ratio in tumor tissues in each treatment group was significantly lower than that in the saline group (*p* < 0.0001), and the ratios of PCNA-positive to total cell in the NIR-treated nano-preparation groups were lower than those in the non-NIR-treated groups (*p* < 0.0001). For the NIR-treated groups, the PTX/IR780-RBCm-LCNP group exhibited the lowest PCNA-positive to total cell ratio among the uncoated nano-preparation groups (*p* < 0.01), and the PCNA-positive to total cell ratio in the PTX/IR780-HA&RBCm-LCNP group was the lowest among the tested groups (*p* < 0.0001). We further found that the induction of tumor cell apoptosis was associated with the Bcl-2/Bax and caspase-3 signaling pathways, and the NIR-treated PTX/IR780-HA&RBCm-LCNPs had the strongest upregulatory effects on caspase-3 in A549 tumor tissue (*p* < 0.0001). These results are in accordance with the results we obtained in the in vitro studies, indicating that PTX and IR780 induce the apoptosis of A549 cells via endogenous mitochondrial pathways. In conclusion, chemotherapy and PDT using PTX and IR780 had remarkable effects on the apoptosis and proliferation inhibition of A549 cells, and the multifunctional HA&RBCm-LCNPs dramatically enhanced these antitumor effects.

In addition, although there was no significant difference in tumor volume and weight between LCNPs + NIR and RBCm-LCNPs + NIR, the RBCm-LCNPs + NIR group had more serious tumor cell apoptosis and less PCNA expression than those of the LCNPs + NIR group (*p* < 0.01). Besides, the expression of Caspase 3 in the tumor tissues of the RBCm-LCNPs + NIR group was significantly higher than that of the LCNPs + NIR group (*p* < 0.0001). These results indicate that RBCm coating enhanced the anti-tumor effect of PTX/IR780-LCNPs.

### Preliminary biosafety analysis

With the exception of nude mice in the PTX Injection group, a slow increase in body weight was observed during the treatment period (Additional file 1: Figure S6). The nano-formulation groups had no appreciable effects on mouse body weight relative to the PTX Injection group.

**Fig. 11:**
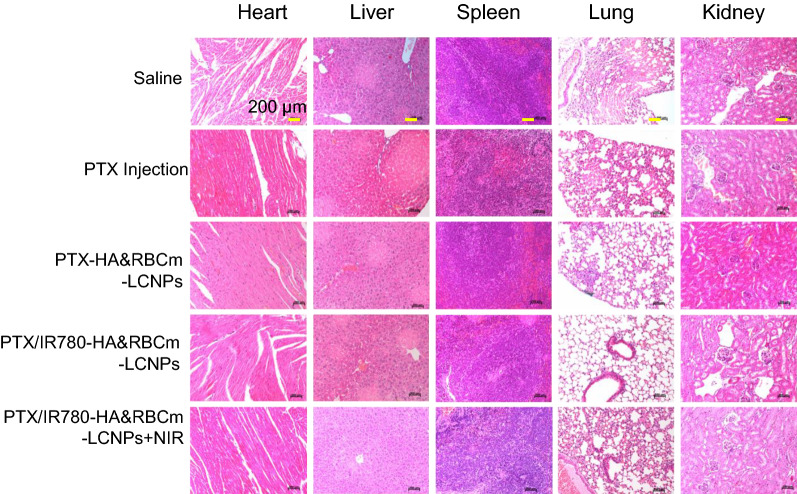
Micrographs of hematoxylin & eosin-stained pathological sections collected at the end of the 13-day treatment period. A549 tumor-bearing BALB/c nude mice were intravenously administered normal saline (Saline, control) and drug-loaded nanoparticle preparations once every 2 days for 12 days. For the phototherapy group, animals were exposed to NIR at 808 nm (1 W/cm^2^) for 5 min 2 h post-drug administration. PTX: 5 mg/kg; IR780: 1 mg/kg (n = 5)

The H&E-stained tissue sections of the liver and lung in the saline group, PTX Injection group, and PTX/IR780-LCNP group displayed multiple acute hepatocyte focal necrosis, small lesions in the lungs, and inflammatory cell infiltration after treatment (Fig. 11). Significant toxicity was not observed in the heart, spleen, and kidney. Multiple acute hepatocyte focal necrosis was also observed in the PTX/IR780-RBCm-LCNP group; however, there were no evident pathological changes in the lung tissue. This indicates that RBCm-LCNPs effectively reduced pathological changes in tissues. Notably, no noticeable pathological changes were present in the tissues of mice in the PTX/IR780-HA&RBCm-LCNP group. The PTX Injection is typically prepared by dissolving paclitaxel in a polyoxyethylene castor oil and ethanol solvent mixture [76]. It has, however, been revealed that polyoxyethylene castor oil can induce various toxic reactions, such as gastrointestinal reactions, allergic reactions, hypotension, nephrotoxicity, cardiotoxicity, and myelosuppression [77]. Nevertheless, our findings confirmed that HA&RBCm-LCNPs have the potential to improve the antitumor efficacy and biosafety of drugs.

## Conclusions

The advantages of RBCs as coated nanoparticle carriers include: (a) a rich source and convenient preparation; (b) escape from the immune system to achieve extended circulation in vivo; (c) good biocompatibility and biodegradability; (d) reduced toxicity when used in certain nano-formulations; and (e) improved stability with reduced aggregation [78]. Although biomimetic nanocarriers functionalized with erythrocyte membranes are potential competitors for chemically modified nanoparticles, the low tumor-targeting capacity of current drug delivery systems remains a limitation for clinical application. Therefore, the introduction of certain ligands and antibodies to confer active targeting is expected to improve targeted drug delivery in cancer therapy. In this study, by simply hybridizing HA and the erythrocyte membrane, the circulation in the blood was prolonged and accumulation in the tumor was enhanced. Moreover, the combination of chemotherapy and PDT mediated by PTX and low-dose IR780 achieved antitumor benefits and good biosafety, which was further significantly improved by co-loading these agents into HA&RBCm-LCNPs. We also found that the combination of PTX and low-dose IR780 may have potential sensitization effects via the PI3K/Akt pathway, which would be conducive to reversing tumor resistance to PTX; however, further experimental verification is needed to explore this aspect of treatment. In addition, the interaction between PTX and IR780, their in vivo distribution, and their pharmacodynamics remain to be characterized. Nonetheless, we believe that the drug delivery system we describe here has the potential for safe and effective applicability in the clinic and should be explored and developed further.

## Materials and methods

### Materials

PTX (API, purity > 99%) and IR780 iodide were purchased from Meilun Biotechnology Co., Ltd. (Dalian, China) and Merck KGaA (Darmstadt, Germany), respectively. PTX Injection (solution) was purchased from Jiangsu Osaikang Pharmaceutical Co., Ltd. (Nanjing, China). HA (240 kDa) was obtained from Freda Pharmaceutical Group Co., Ltd. (Ji’nan, China). Dioleoyl phosphoethanolamine (DOPE) and phosphatidylcholine (Lipoid S100) were purchased from Lipoid GmbH (Ludwigshafen, Germany). HA-DOPE was previously synthesized in our laboratory; the structure verification results are shown in Additional file 1: Figure S7 [79]. Glycerol dioleate was procured from Macklin Inc. (Shanghai, China). Western Blot Kit was purchased from Elabscience Biotechnology Co., Ltd. (Wuhan, China). Cell Counting Kit-8 (CCK-8) and DCFH-DA were obtained from Beyotime Biotechnology (Shanghai, China). SOSG and fetal bovine serum were purchased from ThermoFisher Scientific (New York, NY, USA). Other chemicals (for cell culture and labeling) were purchased from Jiangsu KeyGEN BioTECH Co., Ltd. (Nanjing, China) and Sinopharm Chemical Reagent Co., Ltd. (Shanghai, China). Antibodies are listed in Additional file 1: Table S3.

### Animals and cell lines

Healthy adult male Sprague-Dawley rats (weight, 200 ± 20 g) and male BALB/c nude mice (weight, 18 ± 2 g) were provided by the Experimental Animal Center of Shanghai University of Traditional Chinese Medicine. The experimental animal ethics committee approval numbers are PZSHUTCM8122104 and PZSHUTCM190301003.

Adenocarcinoma human alveolar basal epithelial cells (A549) were provided by the Shanghai Family Planning Research Institute (Shanghai, China). Mouse mononuclear macrophages (RAW264.7) were obtained from the Institute of Biochemistry and Cells, Chinese Academy of Sciences (Shanghai, China).

### Detection of PTX and IR780

The amount of PTX in the samples were determined through ultra-performance liquid chromatography-mass spectrometry (UPLC-MS/MS, ThermoFisher Scientific). Thermo Syncronis C18 column (50 × 2 mm, 1.7 μm) was used at a column temperature of 40 °C. Acetonitrile/0.1% formic acid (68:32, v/v) was used as the eluent at a flow rate of 0.3 mL/min. An electrospray ionization source was used for positive-ion detection. Settings for the remaining parameters are presented in Additional file 1: Table S4. The endogenous substances in biological samples did not interfere with the detection of PTX (Additional file 1: Figure S8), and specificity was deemed good. To assay the biological samples, a linear relationship was established between the peak area and the concentration of PTX with a range of 2–5000 ng/mL (r = 0.9999), with an extraction recovery of more than 90%.

IR780 was detected using a UV-Vis Cary 8454 instrument (Agilent Technologies Inc., Santa Clara, CA, USA) at a wavelength of 780 nm. The IR780 concentration in the range of 0.25–5 µg/mL showed a linear relationship (r = 0.999) with the absorbance, and IR780 was recovered at a rate of > 90%.

### LCNP preparation

Glycerol dioleate (166 mg), Lipoid S100 (86 mg), Tween 80 (75 mg), ethanol (50 mg), PTX (30 mg), and IR780 (6 mg) were mixed and stirred using a magnetic stirrer at 150×*g* for 24 h to obtain a uniform oil phase. The mixture was then slowly added to deionized water (10 mL), with magnetic stirring at 400 rpm for 24 h. A probe ultrasound (195 W) (JY92-IIN, Scientz Biotechnology Co., Ltd., Ningbo, China) was used for 10 min for LCNP formation.

### RBCm-derived vesicle preparation

The preparation of RBCm-derived vesicles is described in Fig. 2A. Briefly, fresh rat blood was centrifuged at 1000×*g* for 10 min. Blood cells were collected and added to phosphate-buffered saline (PBS; pH 7.4). After sufficient hemolysis, cells were centrifuged, and the precipitate was washed with PBS until a colorless supernatant was obtained. The resulting RBCm were resuspended in PBS, extruded with using a mini extruder (Avanti Polar Lipids, Inc., Alabaster, AL, USA), and filtered with 1,000-nm, 800-nm, 400-nm, and 200-nm Track-Etch membranes (GE Healthcare Life Sciences, Buckinghamshire, UK). Samples were then pressed back and forth 10 times to obtain uniform RBCm-derived vesicles, as previously described [71]. HA-DOPE was mixed with RBCm vesicles for 30 min, and excess free HA-DOPE was removed via centrifugation to obtain HA&RBCm.

### RBCm-LCNP and HA&RBCm-LCNP preparation

LCNPs were mixed with RBCm and HA&RBCm, sonicated for 5 min in a water bath, and extruded through 400-nm and 200-nm Track-Etch membranes using a mini extruder. Samples were then pressed back and forth ten times to obtain RBCm-LCNPs and HA&RBCm-LCNPs (Fig. 2A), as previously described [80].

### Drug loading and encapsulation efficiency

The preparation was added to an ultrafiltration tube (molecular weight cut-off: 100 kDa), centrifuged to dryness at 3000×*g*, and washed twice with water. The filtrate was assayed, and the drug amount was recorded as W_f_, while the drug in the nano-formulations was extracted using 9 volumes (v/v) of methanol to determine the total drug amount (W_t_) in the preparation. The amount of lipid excipients was labeled as W_n_. Drug loading and encapsulation efficiency were calculated using Eqs. 1 and 2, respectively, as follows:

1 $${\text{Drug}}\;{\text{loading}} = \frac{{{\text{W}}_{{\text{t}}} - {\text{W}}_{{\text{f}}} }}{{{\text{W}}_{{\text{n}}} + {\text{W}}_{{\text{t}}} }} \times 100\%$$

2 $${\text{Encapsulation}}\;{\text{efficiency}} = \frac{{{\text{W}}_{{\text{t}}} - {\text{W}}_{{\text{f}}} }}{{{\text{W}}_{{\text{t}}} }} \times 100\%$$

### Determination of particle size and zeta potential

The particle size distribution of the nanoparticles was measured at 25 °C using the dynamic light scattering method. Zeta potential was also measured (Nano ZS90; Malvern Panalytical Company, Malvern, UK). Each test was performed in triplicate.

### Transmission electron microscope imaging

The preparation was diluted 60-fold (v/v) with deionized water and dripped onto a copper mesh. The preparation was dyed with 2% phosphotungstic acid for 30 s and observed using a transmission electron microscope (TEM; JEM-2100; JEOL Ltd., Tokyo, Japan).

The internal structures of the prepared nanoparticles were observed using a Tecnai G2 F20 cryo-transmission electron microscope (cryo-TEM; FEI, Hillsborough, OR, USA). The liquid sample was dropped onto the hydrophilized lacey copper mesh and frozen in glacial ethane. Cryo-TEM imaging was performed using a TEM operating at 200 kV.

### Protein detection

Radioimmunoprecipitation assay (RIPA) cell lysis buffer was added to RBCs, RBCm, RBCm-LCNPs, and HA&RBCm-LCNPs. Subsequently, they were lysed on ice for 30 min and vortexed for 10 s every 10 min. After 30 min, the supernatant (total protein solution) was centrifuged at 10,994×*g* for 10 min at 4 °C. Sample loading buffer was added, the samples were mixed well, centrifuged, and boiled for 5 min for protein denaturation. The obtained sample was processed according to the general procedure for SDS-PAGE and western blotting analysis.

### In vitro release

The preparation was added to a dialysis bag (molecular weight cutoff: 14,000 ± 2000 Da), and 15% (w/v) 2-hydroxypropyl-β-cyclodextrin in PBS was used as a release medium to maintain the sink condition during shaking at 37 °C. Samples were collected at different time points; meanwhile, an equal volume of fresh release medium was added. Paclitaxel and IR780 in the samples were subsequently detected.

### Photothermal effects

The test preparation was diluted with water to IR780 final concentration of 20 µg/mL and 60 µg/mL, and 1 mL of each sample was added to each well of a transparent 24-well plate. Pure water was used as the control. Samples were exposed to NIR at 808 nm (1 W/cm^2^) using a laser generator (MDL-FC-808 nm; Inter-Diff Technology Co., Ltd, Shanghai, China). The temperature of the solution was recorded using an infrared ray thermometer (MC-872; OMRON Corporation, Kyoto, Japan). Each experiment was performed independently three times.

### Singlet oxygen detection

Two hundred microliters of pure water (Control) or nanocarrier solution with different concentrations of IR780 and 20 µL of SOSG (50 µM) were added to a 96-well plate and exposed to NIR at 808 nm (1 W/cm^2^). The fluorescence intensity of the solution in each well was detected using a fluorescent microplate reader (Ex/Em = 504 nm/525 nm; Spark 10 M; Tecan, Männedorf, Switzerland). Each experiment was performed three times.

### Stability in the plasma

Each preparation was separately added to an equal volume of rat plasma before incubation at 37 °C. Samples were retrieved at predetermined time points to determine the change in particle size. Each experiment was conducted in triplicate.

### Hemolysis assay

When hemolysis occurs, hemoglobin escapes from the broken red blood cells, and the solution appears red, so hemolysis can be evaluated based on color changes of the solution. Fresh rat blood was collected in a centrifuge tube pretreated with heparin sodium, centrifuged at 1000×*g* for 10 min at 4 °C to retrieve RBCs, which were washed with normal saline and diluted to 2% (v/v) RBC suspension. Equal volume of preparations diluted in normal saline, normal saline (negative control), and 1% Triton X-100 (positive control) were also prepared.

### Nanoparticle uptake by A549 cells

A549 cells were seeded at a density of 1 × 10^5^ cells/well in 6-well plates (for flow cytometry detection; Corning Incorporated, Corning, NY, USA) or in glass-bottom dishes (for imaging; Wuxi NEST Biotechnology, Wuxi, China) and cultured for 24 h before incubation with fresh Dulbecco’s modified Eagle medium containing 10% fetal bovine serum, 1% penicillin and streptomycin (blank control group) or C6-labeled formulation (final concentration of 1 µg/mL diluted in the medium). The mixture was incubated at 37 °C for 2 h. Thereafter, the samples were subjected to the following analyses: (a) the fluorescence intensity of cells was measured using FACS using a flow cytometer (FACS-Canto; Becton, Dickinson and Company, Lake Franklin, NJ, USA); and (b) cells were fixed with 4% paraformaldehyde for 20 min, stained with DAPI for 8 min, and observed using a CLSM (TCSSP8; Leica, Wetzlar, Germany) within 2 h. Ex/Em values were 415 nm/485 nm and 498 nm/568 nm, respectively. Each experiment was carried out in triplicate.

### Cytotoxicity, apoptosis, reactive oxygen species, and tubulin expression assays

A549 cells were seeded at a density of 5 × 10^3^ cells/well in 96-well plates (Corning Incorporated) for the cytotoxicity assay; 2 × 10^5^ cells/well in 6-well plates for apoptosis determination using flow cytometry; and 1 × 10^5^ cells/well in glass-bottom dishes for apoptosis, ROS, and tubulin assays using a CLSM. Cells were cultured at 37 °C in 5% CO_2_ for 24 h and incubated with fresh medium (control group). Each preparation that was diluted with fresh medium (for ROS detection, tubulin analysis, and apoptosis assay, the final PTX and IR780 concentrations were 5 µg/mL and 1 µg/mL, respectively) was added to cells and incubated for 24 h. For the phototherapy group, cells were exposed to NIR at 808 nm (1 W/cm^2^) for 5 min 2 h post-drug administration. Thereafter, the following detections were performed separately:

For the cytotoxicity assay, 10 µL of the CCK-8 reagent was added to each well prior to a 1-h incubation period. Absorbance at a wavelength of 450 nm was measured using a microplate reader. Cell viability was calculated using Eq. 3.

3 $${\text{Cell}}\;{\text{viability}} = \frac{{{\text{OD}}_{{{\text{preparation}}}} - {\text{OD}}_{{{\text{blank}}}} }}{{{\text{OD}}_{{{\text{cell}}}} - {\text{OD}}_{{{\text{blank}}}} }} \times 100 {\% }$$

OD_preparation_, OD_cell_, and OD_blank_ represent the absorbance of cells treated with the test preparations, absorbance of the cells that were not treated with preparations, and absorbance of the medium containing no cells, respectively.

For the apoptosis assay, samples underwent the following processing and corresponding testing: (a) cells were fixed in 4% paraformaldehyde in PBS, then incubated with Hoechst 33342 (5 µg/mL) for 5 min, and observed using a CLSM (Ex/Em = 415 nm/485 nm); (b) cells were digested with EDTA-free trypsin and suspended in 100 µL of binding buffer, and 5 µL of Annexin V-FITC was added to the sample prior to incubation at 4 °C for 30 min. Four hundred microliters of binding buffer and 5 µL of propidium iodide (PI) solution were subsequently added. The mixture was incubated for 5 min at 4 °C and assayed via flow cytometry (FITC, Ex/Em = 490 nm/515 nm; PI, Ex/Em = 535 nm/617 nm). For the ROS assay, cells were incubated with DCFH-DA (10 nmoL/mL) for 30 min and imaged using a CLSM (Ex/Em = 488 nm/525 nm).

For the tubulin distribution assay, cells were fixed in 4% paraformaldehyde in PBS for 20 min and treated sequentially with 3% H_2_O_2_ for 10 min, 10% goat serum for 30 min, anti-α-tubulin antibody (1:1,000) for 90 min, FITC-labeled antibody for 60 min, and DAPI for 10 min. Samples were immediately imaged using a CLSM (Ex/Em = 590 nm/617 nm).

### Cell migration assay

A549 cells were seeded in a 6-well plate at a density of 5 × 10^5^ cells/well and cultured in 5% CO_2_ at 37 °C for 24 h. The culture medium was removed, and the cells were subjected to a cell-scratch test. Two milliliters of incomplete medium (control group) and the preparations, diluted with incomplete medium, containing a final concentration of 2.5 µg/mL paclitaxel and 0.5 µg/mL IR780, were added to the wells. In the phototherapy group, after 2 h of drug administration, cells were exposed to NIR at 808 nm (1 W/cm^2^) for 5 min. Scratches were observed with a microscope (DMi1; Leica) after 0 and 24 h. ImageJ (NIH, Bethesda, MD, USA) was used for statistical analysis of migration distance. Thereafter, cells were collected, and the related proteins were assayed through western blotting. Each experiment was performed in triplicate.

### Immunogenicity test

RAW264.7 cells were seeded at a density of 3 × 10^5^ cells/well; the assays were performed as described in the nanoparticle uptake assays were performed as aforementioned.

### Establishment of xenograft model in nude mice

Each nude mouse received a subcutaneous injection of 5 × 10^6^ cells. Equation 4 was used to calculate the volume of transplanted tumors in nude mice:

4 $${\text{V}} = \frac{{{\text{L}} \times {\text{W}}^{2} }}{2}$$

where V represents tumor volume (mm^3^), and L and W represent long and short tumor diameters (mm), respectively.

### Pharmacokinetics

Test preparations were intravenously injected into rats with PTX and IR780 doses of 5 and 1 mg/kg, respectively. Blood was sampled, and the separated plasma was mixed with a 1:10 volume of docetaxel solution (5 µg/mL; internal standard). Drug was extracted using methyl tert-butyl ether and detected through UPLC-MS/MS. DAS2.0 software was used to calculate pharmacokinetic parameters.

### Label-free quantitative proteomic analysis of protein coronas

The LCNP and HA&RBCm-LCNP solutions were separately incubated with plasma (1:1, v/v) at 37 °C for 1 h, then centrifuged at 6000*×g* for 15 min to obtain nanocarrier-protein complexes. The resulting complexes were washed with PBS to remove unbound proteins, digested with protein lysis buffer, and supplemented with PMSF (1 mM). After shaking for 30 min, the solution was centrifuged at 12,000*×g* for 10 min at 4 °C. Dithiothreitol (5 mM) was mixed with 50 µg of protein from each sample, incubated at 55 °C for 30 min, and cooled to room temperature on ice. Then, iodoacetamide was added to the pellet to a final concentration of 10 mM and incubated in the dark for 15 min. Acetone was added and the mixture was centrifuged at 8000*×g* at 4 °C for 10 min. The precipitate was dissolved in 100 µL of triethylamonium bicarbonate, and proteins were digested with trypsin. The peptides were desalted using a SOLAµ SPE 96-Well Plate (ThermoFisher Scientific), eluted with 60% methanol, and evaporated in vacuo. The samples were analyzed using ThermoFisher Scientific EASY-nLC 1200 and Q Exactive HF systems with a liquid chromatography column (ThermoFisher Scientific Acclaim PepMap RSLC C18; 75 μm × 150 mm, 2 μm, 100 Å). The mobile phase compositions were: phase A, H_2_O-formic acid (99.9:0.1, v/v); and phase B, acetonitrile-H_2_O-formic acid (80:19.9:0.1, v/v/v). The gradient elution procedure was set to 0–82 min, 5–44% B; 82–84 min, 44–90% B; and 84–90 min, 90% B. The mass resolution of the first-order mass spectrometry (MS) was set to 120,000; the m/z was set to 350–1650 for a full scan; and 20 of the main peaks were scanned by MS/MS in positive-ion mode with a resolution of 30,000. Label-free quantification was performed using MaxQuant (version number 1.5.2.8; Max-Planck-Institute of Biochemistry, Planegg, Germany) with Andromeda. The search conditions were controlled by false discovery rate, and the values that did not meet the analytical standards were filtered out using a reverse database and common contaminant database. The specific retrieval parameter settings are listed in Additional file 1: Table S5.

### Biodistribution

Tumor-bearing nude mice with similar body weights and tumor volume were randomly divided into groups. The preparations of paclitaxel and IR780 were intravenously administered at doses of 5 and 1 mg/kg, respectively; normal saline was administered to the control group (saline). Six hours following administration, the blood, heart, liver, spleen, lung, kidney, and tumor tissues were removed and weighed. Tissue samples were mixed three times (m/v) with dimethyl sulfoxide in normal saline (10%, v/v) and homogenized. The homogenate was centrifuged at 4000×*g* for 10 min. Thereafter, 100 µL of the supernatant was taken, and 10 µL of docetaxel solution (5 µg/mL) was added as an internal standard. Subsequent steps were the same as those described in pharmacokinetics assay.

IR780 fluorescence was recorded at predetermined time points using a live imaging system (Ex/Em: 740 nm/804 nm; IVIS Lumina XR; Caliper Life Sciences, Hopkinton, MA, USA) 72 h post-injection. The fluorescence distribution of removed tissues was also imaged.

### In vivo antitumor effects

Tumor-bearing nude mice (tumor volume approximately 50 mm^3^) were randomly assigned to one of the treatment groups or the control group. Mice were intravenously administered PTX/IR780-loaded nanocarriers (5 mg/kg and 1 mg/mL doses of PTX and IR780, respectively) and PTX-loaded preparations (5 mg/kg dose of paclitaxel) once every 2 days for 12 days. For the phototherapy group, mice were exposed to NIR at 808 nm (1 W/cm^2^) for 5 min after 2 h of drug administration. Tumor volume and body weight were measured every 2 days. On the second day following the final administration, the tumor was removed and weighed. Thereafter, the heart, liver, spleen, lung, and kidney were removed. Tissues were fixed with 4% paraformaldehyde for 48 h, embedded in paraffin, and separately subjected to the following assays or staining: (a) hematoxylin & eosin staining; (b) TUNEL assay according to manufacturer’s instructions; and (c) PCNA assay. For PCNA assay, the tissues were sectioned, dewaxed, and subjected to gradient ethanol hydration. The slices were treated with tris-EDTA (1 mmoL, pH 8.0) and H_2_O_2_; incubated with a primary antibody (1:100; PCNA; overnight reaction at 4 °C) and secondary antibody (30 min at 37 °C); and then stained with diaminobenzidine and hematoxylin. Tissue slices were observed via microscopy. ImageJ was used for the semi-quantitative analysis of images. In addition, Bcl-2, Bax, and caspase-3 expression levels in tumor tissues were assayed through western blotting.

### Statistical analysis

One-way analysis of variance was performed using SPSS 19.0 software (IBM, Armonk, NY, USA). A *p* value < 0.05 indicates statistical difference.

## Supplementary Information

**Additional file 1: Figure S1.** Characteristics and blood compatibility of the prepared nano-formulations. **Figure S2.** In vitro release of PTX and IR780 from the PTX/IR780-loaded LCNPs, RBCm-LCNPs, and HA&RBCm-LCNPs (n = 3). **Figure S3.** Characteristics of IR780 in aqueous solution and in nanocarriers. **Figure S4.** In vitro A549 cell viability following treatment with PTX/IR780-loaded RBCm-LCNPs (PTX/IR780-RBCm-LCNPs), and HA&RBCm-LCNPs (PTX/IR780-HA&RBCm-LCNPs) with different concentrations of total PTX and IR780; “+ NIR” refers to near infrared irradiation; the concentration of Blank HA&RBCm-LCNPs is correspond to PTX/IR780-loaded formulations (n = 3). **Figure S5.** In vivo rat plasma concentration-time profiles of PTX following intravenous injection of PTX and IR780 aqueous solution (PTX/IR780-solution), PTX/IR780-loaded LCNPs (PTX/IR780-LCNPs), RBCm-LCNPs (PTX/IR780-RBCm-LCNPs), and HA&RBCm-LCNPs (PTX/IR780-HA&RBCm-LCNPs); the dose of PTX and IR780 were respectively as 5 mg/kg and 1 mg/kg. Data are presented as mean ± SD (n = 6). **Figure S6.** Body weight changes of A549 tumor-bearing Balb/c-nu mice during treatment period. **Figure S7.** FT-IR spectra of HA, DOPE, and HA-DOPE. **Figure S8.** UPLC-MS/MS chromatograms of (A) blank plasma, (B) blank plasma mixed with PTX and docetaxel (DTX), and (C) plasma sample collected from rats following intravenous administration of commercial PTX injection. **Table S1.** Statistics ofadministration methods of IR780 for phototherapy in representative papers published in 2020. **Table S2.** The IC50 of PTX/IR780 preparations against A549 cells in vitro (n = 3). **Table S3.** Antibodies used in the current work. **Table S4.** Mass spectrometry parameter settings. **Table S5.** Retrieval parameters of mass spectrum.

**Additional file 2.** Data of label-free quantitative proteomic analysis of protein coronas.

## Data Availability

The datasets used and analyzed during the current study are available from the corresponding author on reasonable request.
