# Supplementary material for: A novel multi-functionalized multicellular nanodelivery system for non-small cell lung cancer photochemotherapy
Source: J Nanobiotechnology. 2021 Aug 14;19:245. doi: 10.1186/s12951-021-00977-3 (PMC8364713; doi:10.1186/s12951-021-00977-3)
Supplement: Supplementary file 1 — Additional file 1: Figure S1. Characteristics and blood compatibility of the prepared nano-formulations. Figure S2. In vitro release of PTX and IR780 from the PTX/IR780-loaded LCNPs, RBCm-LCNPs, and HA&RBCm-LCNPs (n = 3). Figure S3. Characteristics of IR780 in aqueous solution and in nanocarriers. Figure S4. In vitro A549 cell viability following treatment with PTX/IR780-loaded RBCm-LCNPs (PTX/IR780-RBCm-LCNPs), and HA&RBCm-LCNPs (PTX/IR780-HA&RBCm-LCNPs) with different concentrations of total PTX and IR780; “+ NIR” refers to near infrared irradiation; the concentration of Blank HA&RBCm-LCNPs is correspond to PTX/IR780-loaded formulations (n = 3). Figure S5. In vivo rat plasma concentration-time profiles of PTX following intravenous injection of PTX and IR780 aqueous solution (PTX/IR780-solution), PTX/IR780-loaded LCNPs (PTX/IR780-LCNPs), RBCm-LCNPs (PTX/IR780-RBCm-LCNPs), and HA&RBCm-LCNPs (PTX/IR780-HA&RBCm-LCNPs); the dose of PTX and IR780 were respectively as 5 mg/kg and 1 mg/kg. Data are presented as mean ± SD (n = 6). Figure S6. Body weight changes of A549 tumor-bearing Balb/c-nu mice during treatment period. Figure S7. FT-IR spectra of HA, DOPE, and HA-DOPE. Figure S8. UPLC-MS/MS chromatograms of (A) blank plasma, (B) blank plasma mixed with PTX and docetaxel (DTX), and (C) plasma sample collected from rats following intravenous administration of commercial PTX injection. Table S1. Statistics ofadministration methods of IR780 for phototherapy in representative papers published in 2020. Table S2. The IC50 of PTX/IR780 preparations against A549 cells in vitro (n = 3). Table S3. Antibodies used in the current work. Table S4. Mass spectrometry parameter settings. Table S5. Retrieval parameters of mass spectrum. [file 12951_2021_977_MOESM1_ESM.docx]

**Additional file 1**

**A novel multi-functionalized multicellular nanodelivery system for non-small cell lung cancer photochemotherapy**

**Yongtai Zhang^1#^, Qing Xia^1#^, Tong Wu^1#^, Zehui He^1^, Yanyan Li^1^, Zhe Li^1^, Xuefeng Hou^1^, Yuanzhi He^1^, Shuyao Ruan^1^, Zhi Wang^1^, Jia Sun^2^, Nianping Feng^1^***

*^1^ Department of Pharmaceutical Sciences, Shanghai University of Traditional Chinese Medicine, Shanghai 201203, China*

*^2^ Teaching Experiment Center, Shanghai University of Traditional Chinese Medicine, Shanghai 201203, China*

Fig. S1. Characteristics and blood compatibility of the prepared nano-formulations. A, size distribution and zeta potential ; B, stability in plasma; C, hemolysis assay of PTX/IR780-loaded LCNPs (I), RBCm-LCNPs (II), and HA&RBCm-LCNPs (III) at various total PTX and IR780 concentrations. Data are presented as mean ± SD (n = 3).

Fig. S2. In vitro release of paclitaxel and IR780 from the PTX/IR780-loaded LCNPs, RBCm-LCNPs, and HA&RBCm-LCNPs. (n = 3).

Fig. S3. Characteristics of IR780 in aqueous solution and in nanocarriers. A, ultraviolet-visible light absorption spectrum of IR780 aqueous solution (IR780 solution), IR780-loaded LCNPs, and HA&RBCm-LCNPs; B—D, ultraviolet-visible light absorption spectrum of IR780 formulations by storage in dark and natural light conditions; E, changes of IR780 concentration in nanocarriers solution during exposed to the near infrared light (808 nm, 1 W/cm^2^) for up to 5 min. (n = 3).

Fig. S4. In vitro A549 cell viability following treatment with PTX/IR780-loaded RBCm-LCNPs (PTX/IR780-RBCm-LCNPs), and HA&RBCm-LCNPs (PTX/IR780-HA&RBCm-LCNPs) with different concentrations of total PTX and IR780; “+ NIR” refers to near infrared irradiation; the concentration of Blank HA&RBCm-LCNPs is correspond to PTX/IR780-loaded formulations. (n = 3).

Fig. S5. In vivo rat plasma concentration-time profiles of PTX following intravenous injection of PTX and IR780 aqueous solution (PTX/IR780-solution), PTX/IR780-loaded LCNPs (PTX/IR780-LCNPs), RBCm-LCNPs (PTX/IR780-RBCm-LCNPs), and HA&RBCm-LCNPs (PTX/IR780-HA&RBCm-LCNPs); the dose of PTX and IR780 were respectively as 5 mg/kg and 1 mg/kg. Data are presented as mean ± SD (n = 6).

Fig. S6. Body weight changes of A549 tumor-bearing Balb/c-nu mice during treatment period. Mice were intravenously administered normal saline (Saline, control), PTX-loaded commercial injection (PTX Injection), PTX/IR780-loaded LCNPs (PTX/IR780-LCNPs), RBCm-LCNPs (PTX/IR780-RBCm-LCNPs), and HA&RBCm-LCNPs (PTX/IR780-HA&RBCm-LCNPs) once every 2 days for a total of 6 times (each dose: PTX, 5 mg/kg; IR780, 1 mg/kg). (n = 5).

Fig. S7. FT-IR spectra of HA, DOPE, and HA-DOPE. Characteristic absorption peaks of the hydroxyl group in HA and the carbonyl group in DOPE were 3447.75 cm^-1^ and 1733.37 cm^-1^, respectively. Characteristic absorption peak of the hydroxyl group in HA-DOPE intermediate, HA, was 3422.29 cm^-1^; 1733.37 cm^-1^ was the characteristic absorption peak of the carbonyl group in the ester bond of the original DOPE. These results indicate successful HA-DOPE synthesis.

Fig. S8. UPLC-MS/MS chromatograms of (A) blank plasma, (B) blank plasma mixed with PTX and docetaxel (DTX), and (C) plasma sample collected from rats following intravenous administration of commercial PTX injection.

Table S1. Statistics of administration methods of IR780 for phototherapy in representative papers published in 2020.

| Ref. | Administration | Animals | Dose (IR780) | Vehicle | Treatment |
| --- | --- | --- | --- | --- | --- |
| 1 | i.v | B16F10-bearing mice | 20 mg/kg | Nanoparticles | PDT |
| 2 | i.v | Huh-7-bearing mice | 1.5 mg/kg | MSNs | PTT |
| 3 | i.v | Orthotopic bladder cancer model in mice | 4 mg/kg | Nanoparticles | PTT |
| 4 | i.v | OVCAR-3-bearing mouse | appr. 3 mg/kg | Nanoplatform | PDT |
| 5 | i.v | A549R-bearing mice | 7 mg/kg | Nanoparticles | PTT |
| 6 | i.v | 4T1-bearing mice | 20 mg/kg | Nanoparticles | PDT |
| 7 | i.v | 4T1-bearing mice | 1.3 mg/kg | Nanoparticles | PTT & PDT |
| 8 | i.v | 4T1-bearing mice | appr. 2.5 mg/kg | Nanoplatform | PTT & PDT |
| 9 | i.v | 4T1-bearing mice | 3.06 mg/kg | Nanoparticles | PTT |
| 10 | i.v | 4T1-bearing mice | Anthocephalus cadamba, 125 μg/kg; IR780,375 μg/kg | Nanoliposomes | Combined PDT |
| 11 | Orthotopic injection | 4T1-bearing mice | appr. 1 mg/kg | Ethanol solution | PTT |
| 12 | Intraperitoneal injection | 4T1-bearing mice | 0.675 mg/kg | Polymersomes | PTT |
| 13 | i.v | TRAMP-C1-bearing mice | 2.5 mg/kg | Nanoparticles | PTT & PDT |
| 14 | i.v | Pan 02-bearing mice | 0.5 mg/kg | Liposomes | PTT |

Abbreviations: i.v, intravenous injection; appr., approximately; PTT, photothermal therapy; PDT, photodynamic therapy.

Table S2. The IC50 of PTX/IR780 preparations against A549 cells *in vitro*. (n=3)

| Formulations | IC50 (μg/mL) |
| --- | --- |
| PTX/IR780-HA&RBCm-LCNPs+NIR | 0.14 |
| PTX/IR780-RBCm-LCNPs+NIR | 0.24 |
| PTX/IR780-HA&RBCm-LCNPs | 1.84 |
| PTX-HA&RBCm-LCNPs | 2.00 |

Abbreviations: PTX/IR780-LCNPs, PTX/IR780-loaded LCNPs; PTX/IR780-RBCm-LCNPs, RBCm-coated PTX/IR780-LCNPs; PTX/IR780-HA&RBCm-LCNPs, HA&RBCm-coated PTX/IR780-LCNPs; +NIR, with near infrared irradiation.

Table S3. Antibodies used in the current work.

| Antibody | Source; Cat. No. | Applications |
| --- | --- | --- |
| Recombinant Anti-Bax antibody [E63] | Abcam; ab32503 | WB |
| Recombinant Anti-Bcl-2 antibody [EPR17509] | Abcam; ab182858 | WB |
| Anti-CD47 antibody [EPR4150] | Abcam; ab108415 | WB |
| Anti-PCNA antibody | Abcam;ab92729 | IHC |
| Recombinant Anti-MMP-2 antibody | Abcam; ab92536 | WB |
| TIMP-2 (D18B7) Rabbit mAb | CST; #5738 | WB |
| Recombinant Anti-COX-2 / Cyclooxygenase 2 antibody | Abcam; ab179800 | WB |
| Akt1 (C73H10) Rabbit mAb #2938 | CST; #2938 | WB |
| Recombinant Anti-Keap1 antibody | Abcam; ab227828 | WB |
| Recombinant Anti-AKT1 (phospho S473) antibody | Abcam; ab81283 | WB |
| Anti-alpha Tubulin antibody [DM1A] - Loading Control | Abcam; ab7291 | IF |

Abbreviations: WB, western blot; IHC, immunohistochemistry; IF, immunofluorescence.

Table S4. Mass spectrometry parameter settings.

| MS conditions | Parameter Settings | |
| --- | --- | --- |
|  | Paclitaxel | Docetaxel |
| Spray Voltage | 2500 | 2500 |
| Vaporizer Temperature | 100 | 100 |
| Sheath Gas Pressure | 25 | 25 |
| Aux Gas Pressure | 5 | 5 |
| Capillary Temperature | 320 | 320 |
| Tube Lens | 76 | 89 |
| Collision Energy | 18 | 23 |
| [M+H]^+^ (m/z) | 854.273 | 808.292 |
| Main Fragment Ion Peak (m/z) | 285.8 | 327 |

Table S5. Retrieval parameters of mass spectrum.

| Item | Value |
| --- | --- |
| FDR | 0.01 |
| Missed cleavage | 2 |
| Fixed modification | Carbamidomethyl (C) |
| Variable modification | Oxidation (M) |
| Decoy database pattern | reverse |
| enzyme | Trypsin |
| MS/MS tolerance | 20 ppm |
| MS tolerance | 20 ppm |
| Database | uniprot-proteome_UP000002494-20200817.fasta |
